# Temporal drivers of abundance and community structure of scyphozoan jellyfish in tropical coastal waters

**DOI:** 10.7717/peerj.18483

**Published:** 2025-01-16

**Authors:** Wan Mohd Syazwan, Amy Yee-Hui Then, Ving Ching Chong, Mohammed Rizman-Idid

**Affiliations:** 1 Institute of Biological Sciences, Faculty of Science, Universiti Malaya, Kuala Lumpur, Malaysia; 2 Department of Biology, Faculty of Science, Universiti Putra Malaysia, UPM Serdang, Selangor, Malaysia; 3 Institute of Ocean and Earth Sciences, Universiti Malaya, Kuala Lumpur, Malaysia

**Keywords:** Physicochemical, Assemblage, Diel, Tide, Rainfall, Jellyfish, Lunar

## Abstract

Population blooms of scyphozoan jellyfish in tropical shallow water regions can fuel localized fisheries but also negatively impact human welfare. However, there is a lack of baseline ecological data regarding the scyphozoans in the region, which could be used to manage a fast-growing fishery and mitigate potential impacts. Thus, this study aims to investigate the temporal factors driving the distribution of scyphozoan community along the environmental gradients under different monsoon seasons, rainfall periods, moon phases, and diel-tidal conditions in the Klang Strait located in the central region along the west coast of Peninsular Malaysia, where bloom events are increasing. Scyphozoan samples were collected using commercial bag nets during a 19-month survey. Temporal variations in species abundance and composition were evident and related to the local environmental parameters (salinity, dissolved oxygen, temperature, turbidity, and pH) that varied with the regional monsoon events, although these effects appeared to be species-specific. *Phyllorhiza punctata*, *Acromitus flagellatus*, *Lychnorhiza malayensis*, and *Rhopilema esculentum* were more abundant during the wetter northeast monsoon (NEM) while the abundance of *Chrysaora chinensis* and *Lobonemoides robustus* increased during the drier southwest monsoon (SWM). During the wet period of NEM, scyphozoan abundance was generally higher during the daytime than night-time. The regional monsoon regime and local hydrological events account for jellyfish abundance in the nearshore area with concurrent threats to coastal tourism and power plants, as well as benefits to fisheries especially during the NEM.

## Introduction

Over the past decades, a significant increase in bloom incidents of scyphozoan jellyfish has been reported in marine ecosystems worldwide (Purcell, 2005; Richardson et al., 2009; Lucas & Dawson, 2014). These reports had triggered a series of ongoing debates; one being the magnitude of the reported increases purportedly amplified beyond available evidence in the reviews by Condon et al. (2013) and Pitt et al. (2018). Another pertains to the nature of these blooms –whether they may be ‘true’ blooms, *i.e.,* seasonal and generally predictable population fluctuations, or merely aggregations driven by the environment, which had led to recommendations on the use of terms appropriate for distinct events, *i.e.,* ‘bloom’, ‘swarm’, and ‘aggregation’ (see Graham, Pagès & Hamner, 2001; Hamner & Dawson, 2009; Fernández-Alías, Marcos & Pérez-Ruzafa, 2021). Nevertheless, the percentage of scyphozoan species for which ‘bloom events’ had been recorded has increased from 14% to 25% (Fernández-Alías, Marcos & Pérez-Ruzafa, 2021), clearly demonstrating a growing concern for these developments, given the negative impacts associated with these blooms (Richardson et al., 2009; Lucas & Dawson, 2014; Syazwan et al., 2020).

Jellyfish population distribution and blooms are driven by interactions with many physicochemical parameters such as light (Djeghri et al., 2019), temperature (Loveridge, Lucas & Pitt, 2021; Fernández-Alías et al., 2023), precipitation and river discharge (Banha et al., 2020), salinity (Loveridge et al., 2021), turbulence (Graham, Pagès & Hamner, 2001), tidal state and water currents (Gordon & Seymour, 2009; Fossette et al., 2015), local climate (Lee et al., 2021), weather variability (Gershwin et al., 2014), and nutrient input (Fernández-Alías et al., 2022). Synergistic interactions between environmental conditions, biotic factors, and anthropogenic disturbances have been identified as drivers of jellyfish bloom events in the temperate and sub-tropical waters (Purcell, 2012; Lucas & Dawson, 2014).

Interannual variability and seasonality in abundance are common for many scyphozoan jellyfish, especially for the temperate and sub-tropical species (Suchman et al., 2012; Fleming, Harrod & Houghton, 2013; Kaneda et al., 2013; Rosa et al., 2013; Yoon et al., 2014; Leoni et al., 2021). *Cotylorhiza tuberculata* (Macri, 1778), *Rhizostoma pulmo* (Macri, 1778), and *Aurelia solida* Browne, 1905 exhibited fluctuations in abundance over a decade in Mar Menor, Spain, with major bloom events recorded for *C. tuberculata* and *R. pulmo* during 2011–2012, while *A. solida* blooms concentrated during 2020–2021 (Fernández-Alías et al., 2023). In Lake Illawarra, Australia, *Catostylus mosaicus* (Quoy & Gaimard, 1824) exhibited a seasonal abundance pattern that peaked from February until July, while the co-existing species, *Phyllorhiza punctata* von Lendenfeld, 1884, only occurred between January and April (Pitt et al., 2004). In the sub-tropical southern East China Sea, hydrographic features and environmental conditions had been shown to affect the assemblages of scyphozoans (Lee et al., 2021), pelagic hydrozoans, and siphonophores (Li et al., 2012; Lo, Yu & Hsieh, 2014; Tseng et al., 2015). However, the connection between these factors and jellyfish blooms in tropical regions is poorly understood.

In the tropical Malaysian coastal waters, interactions between temporal environmental changes and coastal populations of fishes, shrimps, mysids, and zooplankton have been reported by various studies (Chew & Chong, 2011; Ramarn, Chong & Hanamura, 2014; Lee, Chong & Yurimoto, 2016; Balqis et al., 2019). However, the temporal variability of jellyfish abundance is relatively understudied despite evident seasonality in population blooms and jellyfish fisheries (Syazwan et al., 2020). With the exception of a commercial jellyfish survey by Rumpet (1991); most of the research on scyphozoans in Malaysia were either preliminary (Kwang, Yahya & Talib, 2009; Chung et al., 2010; Chung, 2012) or short-termed (Rizman-Idid, Farrah-Azwa & Chong, 2016). In-depth understanding of the linkages between environment and jellyfish ecology remains, therefore, unresolved.

On the Malaysian side of the Malacca Strait, scyphozoans are more species-rich and abundant in the conjoint mangrove-mudflat environment in the Klang Strait. The area is one of the major jelly-fishing grounds in Malaysia, where two scyphozoan species, namely *Lobonemoides robustus* Stiasny, 1920 and *Rhopilema esculentum* Kishinouye, 1891, are commercially harvested for local consumption and exported to various Asian countries since the 1970s (Syazwan et al., 2020). Although providing economic benefits *via* the jellyfish fishery, the massive and frequent occurrence of scyphozoan jellyfish (*i.e.,* up to 70 individuals/bag net/hour) in the Strait had interfered with local fisheries activities, power plant operations and coastal tourism (Syazwan et al., 2020). Scyphozoans present envenomation threats and are a common cause of human injury in the Klang Strait. Some local species, particularly *Chrysaora chinensis* Vanhöffen, 1888, *Cyanea* sp., and *Rhopilema hispidum* (Vanhöffen, 1888), can deliver harmful stings with mild symptomatic compilations to humans (Syazwan et al., 2020; Williamson et al., 1996).

To date, no studies have been conducted to determine the seasonality and environmental factors that govern the spatial and temporal abundance of scyphozoan species in the Klang Strait. Given that the hydrographic features of the Klang Strait are highly influenced by the regional monsoon regime and local hydrological events (Chong, King & Wolanski, 2005), this study was conducted to investigate the temporal factors driving the scyphozoan community structure along various physical environmental gradients. This information is essential to mitigate the negative impacts of local scyphozoan blooms on ecosystem and human activities, and can be used to manage and develop jellyfish fishery.

## Materials & Methods

### Study area

**Figure 1:**
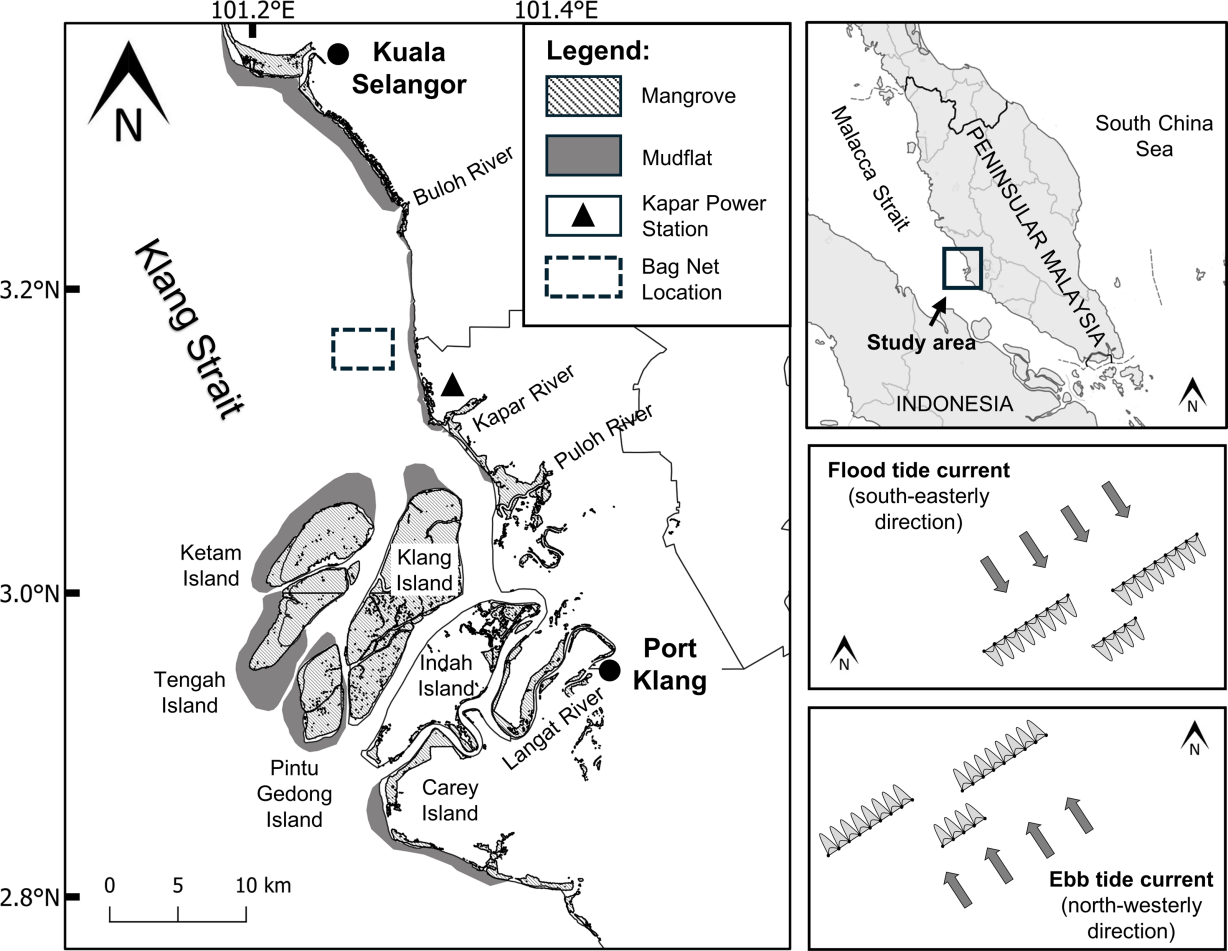
Location map of sampling site (3°10′06.8″N101°16′44.4″E) in the Klang Strait. Sampling sites for monthly and diel samplings are boxed by broken lines and magnified in the right boxes showing the directions of the flood (top box) and ebb (bottom box) tidal streams. Note the arrays of bag nets (maximum of 20) set side by side in three transect lines perpendicularly across the pathway of the tidal stream. The authors originally generated this map using QGIS4.0 and modified using Adobe Illustrator CC 23.0.1.

The narrow Klang Strait, bounded by extensive coastal mudflats, deltaic islands and offshore sandbanks, stretches 70 km into the Malacca Strait from Malaysia’s largest port of Klang (Fig. 1). Extensive mangrove forests (ca. 15,000 ha) currently cover most of the deltaic islands off the Klang River, but on the hinterland agricultural, industrial and other coastal development have extirpated 62% of the original area of 42,723 ha that prevailed in 1960 (Haliza, 2004). The Kapar Power Station (KPS) built on former mangrove forest attests to the burgeoning energy needs of the state to support not only the rapid developments, but also to cater to the needs of the country’s largest concentration of human population in the Klang valley where the capital city of Kuala Lumpur is also located (Chong, King & Wolanski, 2005).

The freshwater inflows into the Strait are mainly from the rivers of Langat, Klang, Buloh, and Selangor. The source of freshwater inputs is attributable to heavy rainfalls during the northeast monsoon (NEM) from November to March, and the inter-monsoon (IN) transition period (April and October). Rainfall during the southwest monsoon (SWM) from May to September is relatively drier when compared to the NEM (Chong, King & Wolanski, 2005; Nelson, 2005).

Marine processes in the area are regulated by primarily two rectilinear tidal currents, the north-westerly ebb (maximum 1.5 m/s) and south-easterly flood currents (maximum 1.0 m/s) (Coleman, Gagliano & Smith, 1970). The spring tidal currents are generally faster (maximum velocity = 1.5 m/s) than the neap tidal currents (maximum velocity = 0.4 m/s). A semi-diurnal ebb and flood cycle occurs over the period of 24 h, with maximum velocities of 1.3 and 1.2 m/s, respectively (Chong, King & Wolanski, 2005). Water depth is generally shallow, ranging from 3.1 to 8.5 m depending on tidal states. The strong tidal current and relatively shallow depths of the Strait are attributable to the increased turbulent mixing of sediments across the water column and the transport of suspended particles along the strait respectively (Chong, King & Wolanski, 2005; Nelson, 2005).

### Field sampling

Jellyfish medusa (*i.e.,* pelagic life stage) were collected at a fixed location (3°10′06.8″N 101°16′44.4″E) about 3.5 km off the mouth of Janggut River (Fig. 1) using commercial stationary bag nets. At low/high slack water, bag nets were deployed side by side in three transects set across the pathway of the tidal stream. This net catches jellyfish, fish and other invertebrates that drift with the flood or ebb tidal run, thus indirectly reflects the direction of their horizontal movement with the respective tide. Each bag net had a mouth opening of approximately 6 m width and height that varied with tidal water height (2–10 m), with net mesh size that ranged from five cm at the mouth to two cm at the cod-end. Based on pilot testing in the study site, the use of the bag net was superior in terms of a wider collection of jellyfish across the whole water column compared to other tested fishing gears such as plankton net, scoop net, trawl net, and gill net (Syazwan et al., 2020).

To assess monthly jellyfish abundance and the effect of monsoon seasonality, samples were collected during daylight flood tide occasion of the full moon on a monthly basis for 19 months (June 2010–December 2011). The sampling duration was fixed at approximately six hours during the flood tide. Collectively, the monthly sampling totaled 19 sampling days, averaging 14 bag nets (replicates) per sampling day (total 272 bag nets).

To investigate the effects of monsoon-driven rainfall (dry-wet) period, moon phases, diel cycle and tidal cycle on scyphozoan abundance, two additional sampling series (henceforth referred to as diel sampling) were conducted on the driest period (June–July, SWM) and wettest period (November–December, NEM) of the year based on past meteorological data (Malaysian Meteorological Department). Jellyfish were collected at approximately 6-hour sampling intervals over a 24-hour cycle weekly for a month during each dry and wet period. The samplings thus covered all daily tides in the tidal cycle (two flood tides and two ebb tides), day and night (diel cycle), and four consecutive moon phases (1^st^ quarter moon, full moon, 3^rd^ quarter moon, new moon). Collectively, the diel sampling totaled eight sampling days with 402 bag nets collected. All jellyfish from the bag nets were instantly examined and photographed in the field for identification using the keys and descriptions of Rizman-Idid, Farrah-Azwa & Chong (2016); Jarms & Morandini (2019), and Syazwan, Low & Rizman-Idid (2020).

To examine the environmental drivers, water parameters were obtained at the surface layer (0.5–1.0 m depth from surface) of the water column during the monthly sampling, and at approximately two hourly-intervals over 24 h during the diel sampling. Parameters including temperature, salinity, pH of water, dissolved oxygen (DO) concentration and turbidity were measured by a pre-calibrated multi-parameter sonde (Hydrolab Datasonde 4a; Hydrolab, Austin, TX, USA), while a pre-calibrated water current meter (Valeport Model 802; Valeport, Devon, UK) was used to measure the water current speed. Water turbidity and current speed readings were collected only during the diel sampling series.

### Data and statistical analysis

#### Monthly rainfall and surface water parameters

In order to establish longer-term rainfall patterns as a function of monsoonal season, daily rainfall data from July 2010 to December 2011 and monthly rainfall data over 12 years (2000–2011) for the nearest meteorological station (MARDI Klang Station), located at the southernmost part of Klang Strait, were obtained from the Malaysian Meteorological Department. The rainfall data were expressed as the standard precipitation index (SPI), *i.e.,* the difference of precipitation from the mean for a specified time period divided by the standard deviation determined from past rainfall records (McKee, Doesken & Kleist, 1993). Monthly SPI for the sampling months (July 2010 to December 2011) was calculated based on the following equation:

\begin{eqnarray*}SPI=(Xi-\bar {X})/SD \end{eqnarray*}

where X*i* is the total rainfall in the *i* th month; $\bar {X}$ and SD are the mean and standard deviation respectively of the monthly rainfall over the 12 year-period (2000–2011). The intensity of rainfall based on the SPI values was categorised into: extremely wet (≥2.00); very wet (1.50 to 1.99); moderately wet (1.00 to 1.49); near normal (−0.99 to 0.99); moderate drought (−1.00 to −1.49); severe drought (−1.50 to −1.99); and extreme drought (≤−2.00) (McKee, Doesken & Kleist, 1993).

Monthly data of rainfall and surface water parameters were pooled by monsoon seasons as SWM (May–September), NEM (November–March) and IN (April and October). Monthly values were summarized using mean and SD and visualized by seasons. One-way ANOVA test was performed on the log-transformed rainfall dataset which satisfied the parametric assumptions, to determine the significant differences between seasons. Tukey HSD test was further conducted for multiple comparisons of the means.

Given that rainfall during the IN season was determined to be insignificantly different from NEM, subsequent monsoonal comparisons pooled data for IN and NEM. Due to the violation of parametric assumptions, the non-parametric Mann–Whitney *U* test was used to compare differences in surface water parameters between monsoon seasons (SWM/NEM+IN). For diel data, non-parametric Kruskal–Wallis ANOVA and Mann–Whitney U tests were performed to examine the effects of rainfall periods, moon phases, diel cycle, and tide cycle on surface water parameters.

### Monthly and diel jellyfish assemblage

Jellyfish relative abundance (henceforth referred to as abundance) based on catch per unit effort (CPUE) was computed based on the total number of individuals collected per bag net. Kruskal–Wallis ANOVA and Mann–Whitney *U* tests were performed to examine if moon phase, diel cycle, and tide cycle had significant effect on total jellyfish abundance. All univariate statistical analyses (significance level at *α* = 0.05) were computed using the Statistica Version 9 software package (StatSoft, Tulsa, OK, USA).

For subsequent multivariate analyses, abundance data was subjected to square root transformation. A Bray–Curtis resemblance matrix was created from the transformed data. Distance-based permutational multivariate analysis of variance (PERMANOVA) test was used to compare the assemblage between monsoon seasons for monthly data. To simplify interpretation of the diel data, the temporal variability in jellyfish assemblage (abundance and species composition) was compared amongst and within two nested factors (moon phase nested within dry-wet periods, and tidal cycle nested within diel cycle) using PERMANOVA. The PERMANOVA test was performed with Type III (partial) sums of squares using 4999 permutations of residuals under a mixed model (Anderson, Gorley & Clarke, 2008; Anderson & Legendre, 1999; Anderson & Braak, 2003). PERMANOVA was followed by the *post-hoc* pairwise test if the comparison amongst and within temporal variables was significant (*p* < 0.05).

Similarity percentages (SIMPER) analysis was performed on the transformed data to identify significant species that contribute to the dissimilarity between assemblages, and similarity within assemblages for all factors and their combinations. Species that fulfilled the criteria % *δ*_*i*_ >3% and *δ*_*i*_ = SD >1 (where *δ*_*i*_ is the overall dissimilarity (or similarity) between (or within) groups *i* and SD is the standard deviation) were arbitrarily accepted as important contributors to dissimilarity or similarity among/within each factor.

### Association between water parameters and diel jellyfish assemblage

The effect of water parameters on assemblage structure was assessed by using distance-based linear models (DistLM). Prior to DistLM, draftsman plots and correlation matrices were produced from the untransformed mean value of environmental data matrix, to assess the normality and collinearity among variables. From the draftsman plot, turbidity was right-skewed, and therefore was log (x)-transformed to normalize the distribution, while other variables were untransformed. Collinearity effect (*r* > 0.95) among variables was not detected, and therefore all six variables (temperature, salinity, pH, (log) turbidity, DO concentration and water current speed) were retained for subsequent DistLM procedures. DistLM was performed by grouping the variables with selection based on the adjusted R^2^ criterion, step-wise selection procedure and 4999 permutations (Anderson, Gorley & Clarke, 2008).

The constrained distance-based redundancy analysis (dbRDA) plots were used to visualize the most parsimonious model indicated by DistLM results. Jellyfish samples were grouped based on centroids of assemblage, and vectors for individual environmental variable proportional to their contribution to the total variation were displayed. Additionally, linear relationships between individual environmental parameters based on fitted model with dbRDA coordinate axes (multiple partial correlations: *r* > 0.2) were assessed. All multivariate routines (PERMANOVA, SIMPER, DistLM) were performed in the statistical software package PRIMER 6 (Clarke & Gorley, 2006) with PERMANOVA+ (Anderson, Gorley & Clarke, 2008).

## Results

### Rainfall data and monsoon seasons

**Figure 2:**
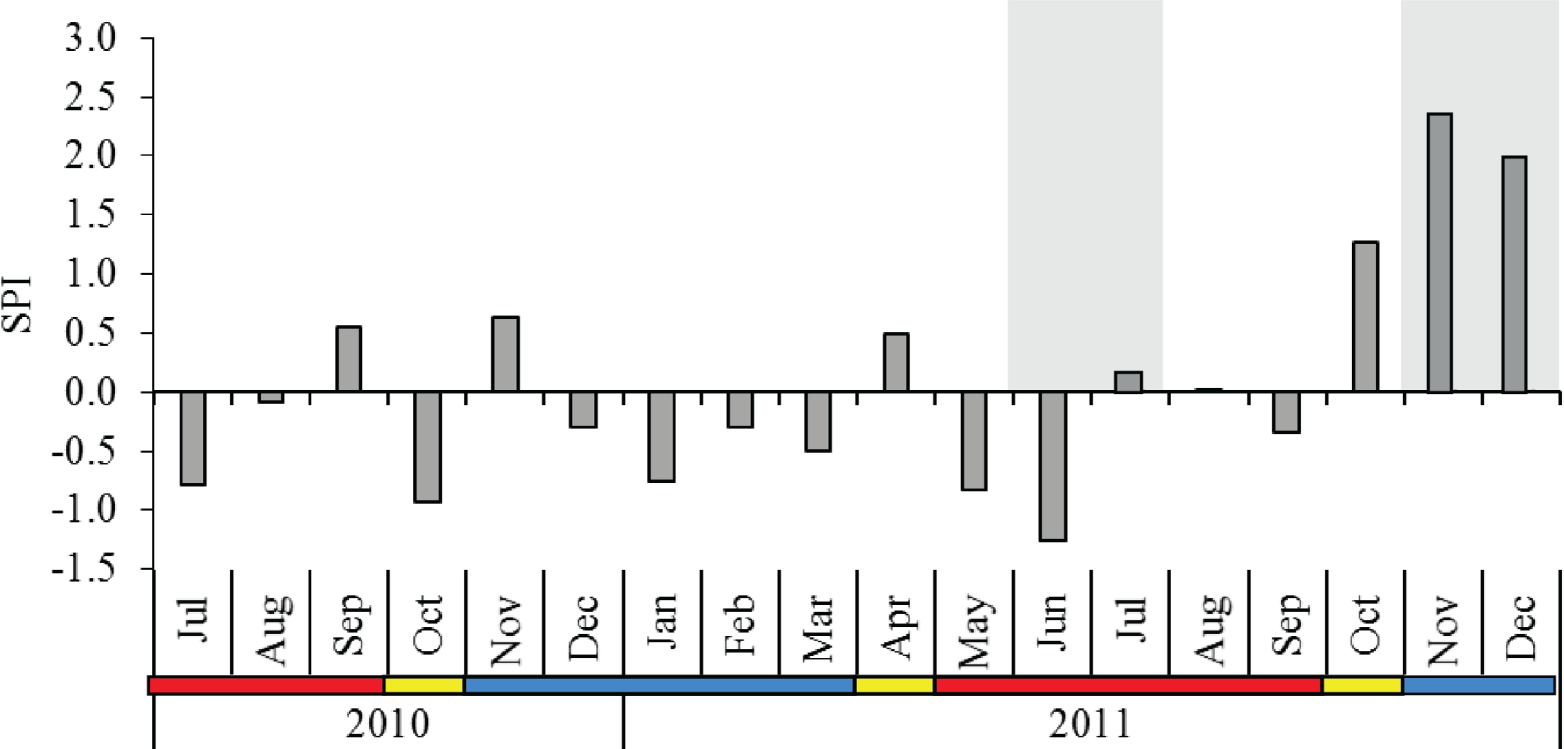
Monthly standardized precipitation index (SPI) in Klang Strait from July 2010 to December 2011. Shaded grey area at the background indicate the periods of diel sampling. Data obtained from the Malaysian Meteorological Department. Blue horizontal bar = northeast monsoon (NEM); red horizontal bar = southwest monsoon (SWM); yellow horizontal bar = inter-monsoon (IN).

The 12-year rainfall pattern was significantly different between the monsoon seasons (*p* < 0.001), and *post-hoc* comparison indicated that SWM showed significantly lower rainfall than IN and NEM (Table 1). The SPI from July 2010 to December 2011 averaged 0.08 with a moderately wet period (SPI = 1.28) recorded during IN season in October 2011. Extreme wet periods (SPI = 2.37 in November 2011, SPI = 1.97 in December 2011) occurred during the NEM (Fig. 2). No severe drought seasons (SPI <−1.5) were observed throughout the sampling months, with the exception of a ‘moderate drought’ in June 2011 (SPI = −1.26) during SWM. The diel sampling series coincided with the dry (moderately-drought) period in SWM, and November–December 2011 during the extremely-wet period in NEM (Fig. 2).

**Table 1:** Descriptive statistics and results of one-way ANOVA and post-hoc Tukey HSD tests on mean rainfall data among monsoon seasons over 12-year period (2000 to 2011).

**Monthly total**	**Season**			**p-level**
**rainfall (mm)**	**SWM**	**IN**	**NEM**	
$\bar {x}$	141.9^a^	288.1^b^	244.3^b^	0.000^**^
±SD	76.5	122.4	104.9	
Min	32.5	104.2	86.4	
Max	406.3	540.4	570.6	
n	57	24	60	

**Notes.**

Homogenous groups indicated by superscripts a and b.

SWM southwest monsoon NEM northeast monsoon IN inter-monsoon $\bar {x}$ mean SD standard deviation Min minimum Max maximum n no. of replicates

** Significance at *p* < 0.001.

### Monthly variations in surface water parameters

**Figure 3:**
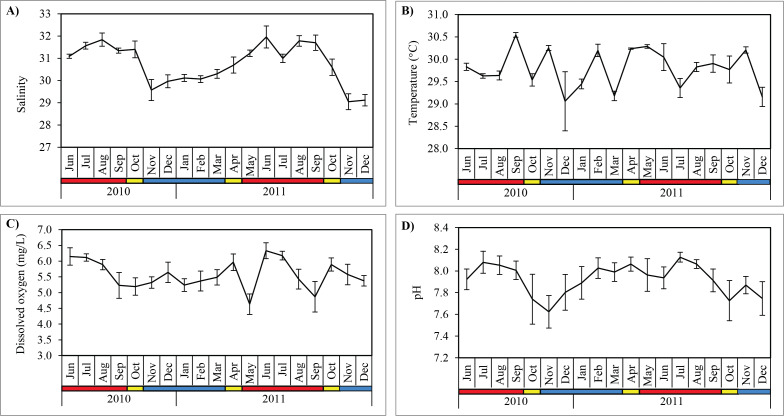
Monthly mean values and standard deviations of surface water parameters recorded at sampling site in Klang Strait from June 2010 to December 2011. (A) Salinity. (B) Temperature. (C) Dissolved oxygen. (D) pH. Blue horizontal bar = northeast monsoon (NEM); red horizontal bar = southwest monsoon (SWM); yellow horizontal bar = inter-monsoon (IN).

Salinity was significantly higher (Mann–Whitney U; *p* < 0.001) during SWM (31.5 ± 0.4) compared to NEM and IN (30.1 ± 0.7) (Table 2; Fig. 3). Water temperature was the highest in September 2010 (30.1 ± 0.1 °C) during the SWM, while the lowest mean temperature occurred in December 2010 (29.1 ± 0.7 °C) during NEM (Fig. 3), although the differences between season was not significant (*p* = 0.114) (Table 2). Dissolved oxygen (DO) concentrations averaged 5.6 ± 0.5 mg/L and were highly variable during SWM months (Table 2; Fig. 3) but were not significantly different between seasons (*p* = 0.119). Surface pH values during SWM months (8.0 ± 0.1) were significantly greater (*p* = 0.001) than in NEM and IN (7.8 ± 0.2) (Fig. 3).

**Table 2:** Descriptive statistics and results of Mann–Whitney *U* tests on environmental parameters and scyphozoan abundance (CPUE) between monsoon seasons (monthly sampling routine) and rainfall periods (diel sampling series) in Klang Strait.

		**Monsoon Season**		***p*-level**	**Rainfall Period**		***p*-level**
		**SWM**	**NEM+IN**		**Dry**	**Wet**	
***Water Paramater***	n	27	30		89	81	
Salinity	$\bar {x}$	31.5	30.1	0.000^**^	31.1	28.8	0.000^**^
	±SD	0.4	0.7	SWM>NEM+IN	0.4	0.7	Dry>Wet
Temperature (°C)	$\bar {x}$	29.9	29.7	0.114	30.0	29.6	0.000^**^
	±SD	0.4	0.5		0.3	0.4	Dry>Wet
Dissolved Oxygen (mg/L)	$\bar {x}$	5.6	5.5	0.119	5.4	4.2	0.000^**^
	±SD	0.7	0.3		0.7	0.4	Dry>Wet
pH	$\bar {x}$	8.0	7.8	0.001^*^	7.8	7.8	0.065
	±SD	0.1	0.2	SWM>NEM+IN	0.1	0.2	
Turbidity (NTU)	$\bar {x}$	–	–	–	135.3	145.1	0.083
	±SD	–	–		204.8	219.6	
Water Current (m/s)	$\bar {x}$	–	–	–	0.4	0.4	1.000
	±SD	–	–		0.1	0.2	
***Jellyfish Abundance***	n	124	148		217	185	
*Phyllorhiza punctata*	$\bar {x}$	1.4	6.4	0.000^**^	0.3	7.8	0.000^**^
	±SD	2.4	6.1	SWM<NEM+IN	0.6	5.3	Dry<Wet
*Cyanea* sp.	$\bar {x}$	2.9	3.0	0.060	3.0	2.9	0.109
	±SD	2.3	3.6		3.0	3.7	
*Lobonemoides robustus*	$\bar {x}$	2.9	0.4	0.000^**^	0.5	0.5	0.977
	±SD	3.9	0.9	SWM>NEM+IN	0.7	0.9	
*Rhopilema esculentum*	$\bar {x}$	0.1	0.7	0.000^**^	0.1	1.0	0.000^**^
	±SD	0.3	1.2	SWM<NEM+IN	0.3	1.2	Dry<Wet
*Rhopilema hispidum*	$\bar {x}$	0.1	0.1	0.794	0.1	0.1	0.562
	±SD	0.4	0.3		0.2	0.2	
*Chrysaora chinensis*	$\bar {x}$	0.1	0.2	0.527	0.2	0.1	0.033^*^
	±SD	0.3	0.5		0.6	0.3	Dry>Wet
*Lychnorhiza malayensis*	$\bar {x}$	0.0	0.1	0.362	1.4	0.8	0.000^**^
	±SD	0.2	0.6		8.1	1.4	Dry>Wet
*Acromitus flagellatus*	$\bar {x}$	0.0	0.1	0.398	0.1	0.2	0.001^**^
	±SD	0.1	0.3		0.3	0.7	Dry<Wet
All species	$\bar {x}$	7.5	10.9	0.000^**^	5.5	13.3	0.000^**^
	±SD	4.2	7.0	SWM<NEM+IN	8.4	8.1	Dry<Wet

**Notes.**

SWM southwest monsoon NEM northeast monsoon IN inter-monsoon $\bar {x}=$ mean SD standard deviation n sample size (no. of replicate) = data unavailable

* Significance at *p* < 0.05.

** Significance at *p* < 0.01.

### Monthly scyphozoan composition and abundance

A total of 2,707 individual scyphozoan jellyfish from eight species were collected during the monthly sampling. The order Rhizostomae was the most diverse group, represented by six species (from five families), namely *P. punctata*, *L. robustus*, *R. esculentum*, *R. hispidum*, *Lychnorhiza malayensis* Stiasny, 1920 and *Acromitus flagellatus* (Maas, 1903). The order Semaestomeae constituted two species (from two families); *Cyanea* sp. and *C. chinensis*. *P. punctata* was numerically the most dominant species, comprising 41.5% of the total monthly catch, followed by *Cyanea* sp. (29.5%)*, L. robustus* (21.1%) and *R. esculentum* (4.0%). Other species altogether represented <4% of total jellyfish catch.

The PERMANOVA results indicated that the scyphozoan community was significantly different between monsoon seasons (*p* < 0.001, pseudo-F = 53.82), with higher overall abundance during NEM and IN months (mean CPUE = 10.9 ± 7.0), compared to SWM (mean CPUE = 7.5 ± 4.2) (Table 2). Pairwise tests indicated that *P. punctata* made the highest contribution (33.7%) to the dissimilarity in assemblage between seasons, followed by *Cyanea* sp. (23.1%) and *L. robustus* (22.6%) (Table 3). These results reflected the monthly variations in species abundance discussed below.

**Table 3:** Pairwise tests for PERMANOVA and percentage contributions of scyphozoan species to observed dissimilarities (SIMPER) at all levels between monsoon season (SWM/NEM+IN), and within and across two nested factors (nested factors in brackets), namely period(moon) and diel(tide).

				**% of species contribution to dissimilarity**							
**Source of variation**	**Pairwise comparison**	***p*-level**	**Average dissimilarity**	**CY**	**PP**	**LR**	**RE**	**RH**	**CH**	**AF**	**LM**
Monsoon	SWM x NEM+IN	0.000	60.4	**23.1**	**33.7**	**22.6**	10.4	3.4	3.0	1.3	2.4
Period(Moon)	Dry(Neap) x Dry(Spring)	0.002	57.1	**36.2**	16.0	17.7	2.8	3.1	11.1	2.3	10.8
	Dry(Neap) x Wet(Neap)	0.000	71.2	**18.6**	**37.0**	11.0	**17.0**	2.3	4.5	0.8	8.8
	Dry(Neap) x Wet(Spring)	0.000	66.8	**20.3**	**42.9**	8.5	8.5	2.1	5.0	3.4	9.4
	Dry(Spring) x Wet(Neap)	0.000	73.3	**20.6**	**36.6**	10.6	**15.2**	1.2	4.1	1.8	9.9
	Dry(Spring) x Wet(Spring)	0.000	66.9	**18.5**	**43.6**	9.0	8.4	1.2	4.6	4.0	10.8
	Wet(Neap) x Wet(Spring)	0.000	43.4	**25.4**	**22.0**	12.4	**17.1**	1.9	3.4	4.7	13.2
Diel(Tide)	DF x DE	0.000	61.5	**22.0**	**28.9**	12.2	10.9	1.8	5.8	4.5	13.9
	DF x NE	0.000	57.1	**24.0**	**33.2**	14.1	10.2	1.5	7.1	2.4	7.6
	DE x NF	0.000	61.6	**24.8**	**28.2**	11.9	9.9	2.2	5.0	3.9	14.1
	DE x NE	0.000	62.0	**21.2**	**29.1**	9.2	10.4	2.3	6.9	5.2	15.7
	NF x NE	0.000	58.2	**27.6**	**32.4**	13.4	8.8	1.9	6.0	1.6	8.3
Period(Moon)	**Dry(Neap)**										
x	DF x DE	0.022	52.2	**36.3**	21.1	21.3	0.0	5.0	11.7	0.0	4.6
Diel(Tide)	**Dry(Spring)**										
	DF x DE	0.000	68.2	**35.5**	6.7	16.2	6.4	1.3	9.4	6.4	18.1
	DF x NE	0.000	45.9	**34.4**	14.4	**24.9**	2.3	1.9	13.7	0.9	7.4
	DE x NF	0.000	69.2	**40.0**	6.8	**15.8**	6.0	1.2	7.7	5.7	16.8
	DE x NE	0.000	65.2	**28.0**	7.6	11.2	7.7	1.4	13.2	8.1	22.9
	NF x NE	0.000	45.3	**37.3**	14.3	**25.1**	2.8	1.8	11.2	0.8	6.8
	**Wet(Neap)**										
	DF x DE	0.000	42.3	**15.9**	**30.2**	**15.9**	**16.7**	1.2	2.6	2.6	**14.9**
	DF x NE	0.000	43.3	**17.6**	**19.0**	**17.5**	**29.3**	2.7	2.4	2.8	8.8
	DE x NF	0.000	44.2	**15.4**	**32.4**	**15.2**	**16.8**	1.2	2.6	1.2	**15.2**
	DE x NE	0.001	49.3	**16.7**	**21.9**	13.2	**21.3**	3.7	4.6	2.2	**16.3**
	NF x NE	0.001	39.6	**19.5**	**22.1**	17.2	**19.8**	3.3	2.8	1.4	14.0
	**Wet(Spring)**										
	DF x DE	0.001	41.4	**26.7**	**21.0**	9.8	**10.9**	2.7	4.2	7.7	**17.0**
	DE x NF	0.000	35.9	**24.5**	**18.9**	11.3	**12.3**	3.1	2.9	7.8	**19.2**
	DE x NE	0.000	41.5	**27.5**	**20.9**	7.4	**12.2**	2.2	4.5	8.5	**16.8**
	NF x NE	0.000	38.2	**24.7**	**26.5**	11.8	**16.6**	1.2	3.1	5.0	11.1

**Notes.**

Only results with significant effects (PERMANOVA: *p* < 0.05) are shown. Species significant contributions (% *δ*i >3%, *δ*i/SD >1) to dissimilarity are in bold.

SWM southwest monsoon NEM+IN inter- and northeast monsoon Dry dry period Wet wet period Neap neap tide (1^st^ quarter and 3^rd^ quarter moon) Spring spring tide (full and new moon) D day N night F flood tide E ebb tide species CY *Cyanea* sp. PP *Phyllorhiza punctata* LR *Lobonemoides robustus* RE *Rhopilema esculentum* RH *Rhopilema hispidum* CH *rysaora chinensis* AF *romitus flagellatus* LM *Lychnorhiza malayensis*

**Figure 4:**
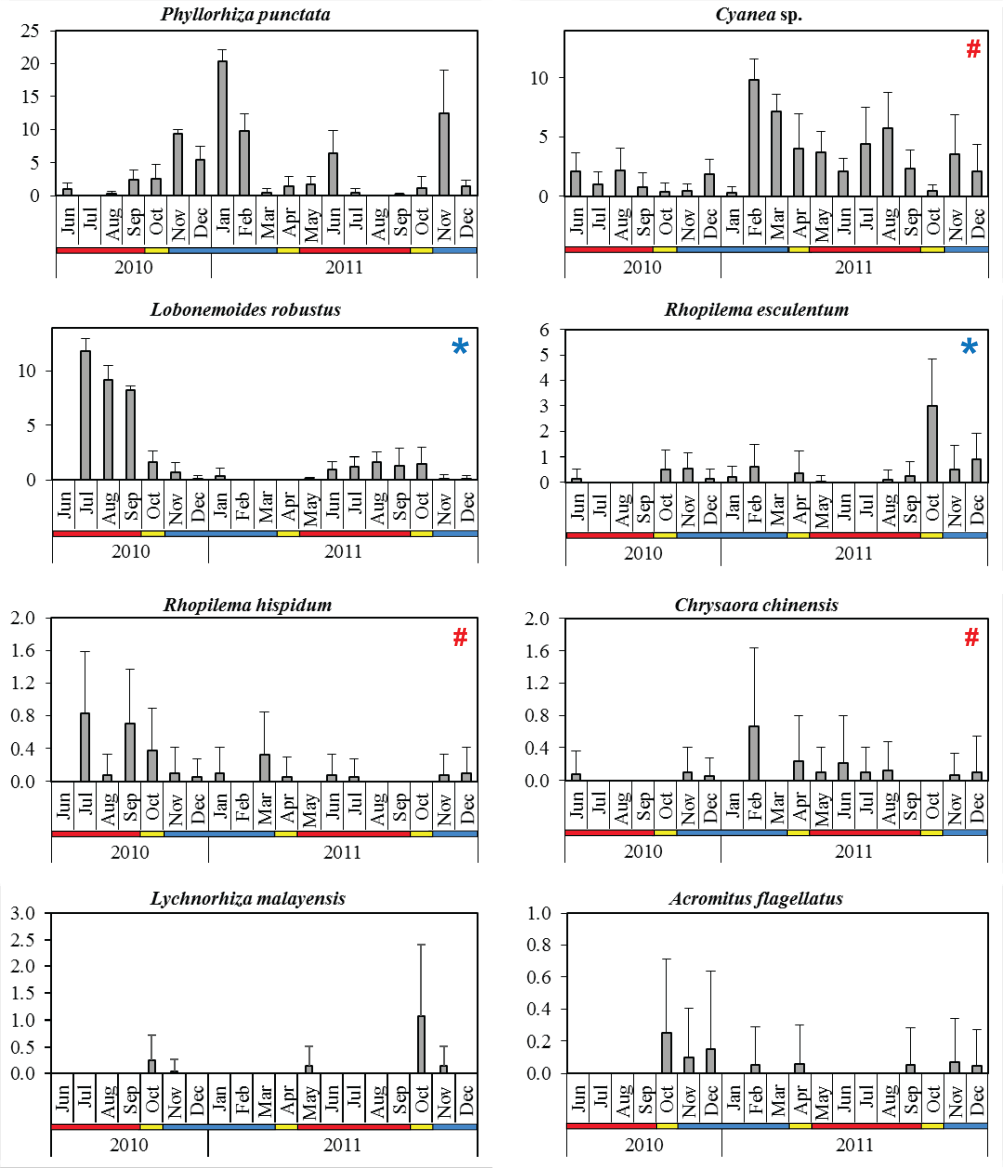
Monthly mean abundance (CPUE) of eight scyphozoan species recorded in the Klang Strait from June 2010 to December 2011. Error bars = standard deviation; blue horizontal bar = northeast monsoon (NEM); red horizontal bar = southwest monsoon (SWM); yellow horizontal bar = inter-monsoon (IN); # = harmful (causing human injury) species; * = commercial (edible) species.

The dominant species *P. punctata* occurred in significantly higher abundance during the NEM and IN (Table 2). Multiple abundance peaks were recorded for this species during November 2010, January 2011, June 2011, and November 2011, although they were absent in July 2010 and August 2011 during the SWM (Fig. 4). The lowest total abundance was observed in June 2010 (CPUE = 3.3 ± 2.5) during the SWM. *Cyanea* sp. was the only scyphozoan present all year round, but their abundance was not significantly different between monsoon seasons (*p* = 0.060, Table 2). Multiple peaks of abundance, were observed for this species particularly in February, August, and November 2011 (Fig. 4), with contributions of 44.2 to 89.6% to the monthly total abundance.

The edible species *L. robustus* occurred during two periods: July 2010 - January 2011 and May - December 2011 (Fig. 4), with peaks recorded in the SWM, particularly in July 2010 (CPUE = 11.8 ± 1.2). However, its numbers significantly decreased during NEM and IN (*p* < 0.001) (Table 2). Another edible species, *R. esculentum* was abundant throughout the NEM and IN season, particularly during October 2010–February 2011 and August–December 2011 (Fig. 4). The highest abundance was recorded in October 2011 (IN season), when the species dominated 41.7% of total species abundance (Fig. 4). *R. esculentum* was significantly less abundant during SWM (*p* < 0.001) (Table 2).

*L. malayensis* occurred on three distinct occasions throughout the sampling duration; October–November 2010, May 2011, and later during October–November 2011 (Fig. 4). Its annual peak of abundance was recorded during IN season, specifically in October 2010 (CPUE = 0.3 ± 0.5) and later in October 2011 (CPUE = 1.1 ± 1.4). *C. chinensis* abundances were not significantly different between monsoon seasons (*p* = 0.527; Table 2), with the highest abundance recorded in February 2011 (CPUE = 0.7 ± 0.97) during the NEM (Fig. 4). *R. hispidum* was similarly abundant across monsoon seasons (*p* = 0.794), particularly in July 2010 (CPUE = 0.83 ± 0.75), September 2010 (CPUE = 0.70 ± 0.68), and March 2011 (CPUE = 0.33 ± 0.51) (Fig. 4). *A. flagellatus* occurred mainly during NEM and IN months; during October–December 2010, February, April, November, and December 2011, with the maximum abundance (CPUE = 0.25 ± 0.46) observed in October 2010 (IN season) (Fig. 4).

### Effect of rainfall period and diel cycle

A total of 3,659 scyphozoan individuals comprising eight species were recorded during the diel sampling series. Total scyphozoan abundance was significantly greater (Mann–Whitney U; *p* < 0.001) during wet than the dry period. Scyphozoan abundance differed significantly among moon phases. In the dry period, total scyphozoan abundance was significantly lower at the 1^st^ quarter moon compared to other moon phases (Kruskal–Wallis ANOVA; *H* = 36.44; *p* = 0.000). As for the wet period, scyphozoan was mostly abundant at the new moon, but its abundance was lower at other moon phases (*H* = 49.73; *p* = 0.000). Total scyphozoan abundance in the dry period was not significantly different (Mann–Whitney U; *p* = 0.096) between diel cycle. However, in the wet period, total species abundance was significantly higher in day-time compared to night-time (*p* = 0.001). Scyphozoan was significantly greater in abundance during flood than ebb tide (*p* < 0.001) in the dry period. Conversely, during the wet period, their abundance was not significantly different between the tides (*p* = 0.226).

The PERMANOVA results indicated that the scyphozoan community was significantly different for the nested factors ‘period(moon)’ (*p* = 0.0002, pseudo-F = 65.99) and ‘diel(tide)’ (*p* = 0.0002, pseudo-F = 7.74). Interaction effects between these two nested factors were significant (*p* = 0.0002, pseudo-F = 4.32), and pairwise comparisons among levels including the nested effects indicated that most of the differences were seen in the dry(spring) and wet(neap) combinations, and some of the wet(spring) combinations (Table 3).

Among the species, *Cyanea* sp. and *P. punctata* were consistently the most significant contributors (% *δ*i >3%, *δ*i/SD >1) to the observed community dissimilarities especially within the levels of ‘period(moon)’ and ‘diel(tide)’ (SIMPER; Table 3). Other species played more important role in driving dissimilarities in communities in different ‘period(moon)’ and ‘diel(tide)’ interactions, especially *L. robustus, R. esculentum,* and *L. malayensis* (SIMPER; Table 3).

*Cyanea* sp. largely dominated the scyphozoan community in the dry period particularly during the day-time of neap tide and night-time of spring tide, with 61.2–92.0% contribution to community similarity (SIMPER; Table S1). Scyphozoan community in the wet period was primarily dominated by *P. punctata,* making up 34.4–71.5% of the community composition across all levels, followed by *Cyanea* sp. that showed higher abundance during spring (25.0–30.8%) compared to neap tide (6.6–18.9%). *R. esculentum* contributed significantly to the wet period community during the day-time of neap tide (25.3–27.6% in contribution) (Table S1).

### Effect of environmental parameters on community structure

The marginal tests in the DistLM analysis on individual environmental parameters (Table 4(A)) indicated that salinity, DO concentration and temperature were significant (*p* < 0.01) in explaining the temporal variation in community structure, with each of them explaining 42.3%, 42.1% and 19.8% of the variations respectively. The subsequent sequential test (adjusted R^2^ criterion) using the stepwise selection procedure is shown in Table 4(B). Temperature, salinity, (log) turbidity, and DO concentration together contributed to 55.4% of the total explained variation in the best explanatory stepwise model (adjusted *R*^2^ = 0.488) based on the sequential test (see Table 4(C)). pH and water current were excluded from the model because the addition of these variables did not significantly increase the explained sum of squares.

**Table 4:** Results of distance-based linear model (DistLM). (A) Results of the marginal tests showing the influence of each environmental parameter in isolation to the differentiation in scyphozoan communities. (B) Results of the sequential tests showing the effect of environmental parameters in the combined model (stepwise selection with adjusted R^2^ criterion). (C) Best solution for the model.

(A) Marginal tests for stepwise model			
Parameter	Pseudo-F	*P*	Explained proportion
Salinity	22.03	**0.000**	0.42
DO	21.86	**0.000**	0.42
Temperature	7.42	**0.001**	0.20
(Log) turbidity	1.49	0.199	0.05
Water current	0.83	0.441	0.03
pH	0.31	0.842	0.01

(B) Sequential tests for stepwise model						
Parameter	Adjusted R^2^	Sum of square	Pseudo-F	*P*	Explained proportion	Cumulative proportion
+Salinity	0.40	14,817.0	22.03	**0.000**	0.42	0.42
+(Log) turbidity	0.46	2,414.6	3.94	**0.010**	0.07	0.49
+DO	0.47	1,158.1	1.95	0.109	0.03	0.53
+Temperature	0.49	999.3	1.73	0.146	0.03	0.55

(C) Sequential tests for stepwise model			
Adjusted R^2^	R^2^	RSS	Selected variable
0.48798	0.5541	15,606	Salinity, DO concentration, temperature, (log) turbidity

**Notes.**

Significant parameters (*p* < 0.05) are highlighted in bold.

DO, dissolved oxygen concentration.

**Figure 5:**
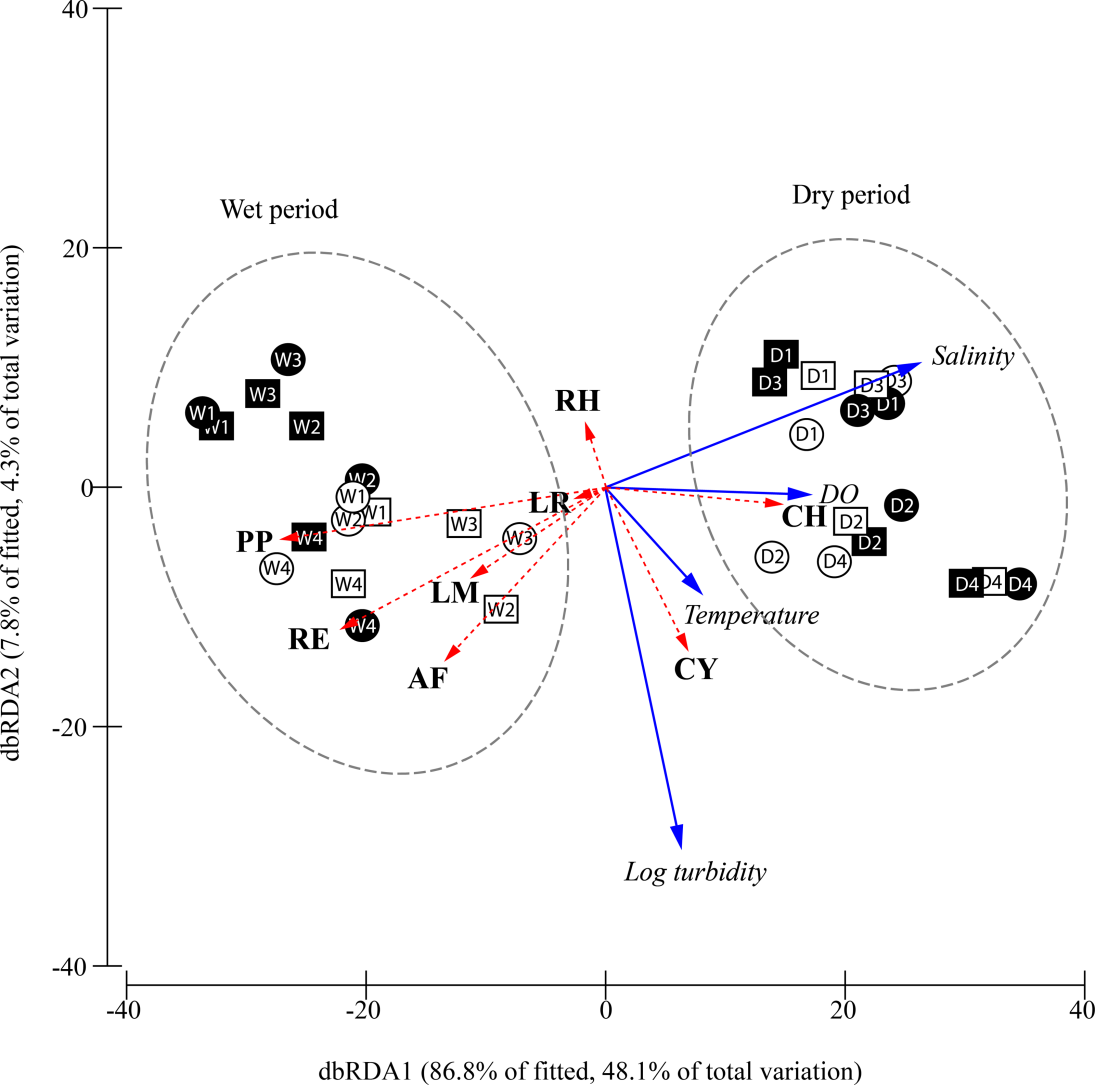
Constrained plot of distance-based redundancy analysis (dbRDA) illustrating the DistLM model derived from Bray–Curtis similarity matrix (distances among centroids) of the square-root transformed species abundances (CPUE) over different periods, moon phase. The effect of the fitted environmental parameters (multiple partial correlation) is illustrated on the plot as vector overlays (solid blue arrows). Individual species contribution to differentiation among factors is indicated by vector overlays (dashed red arrows) based on Spearman correlation. D, dry period; W, wet period; 1, 1^st^ quarter moon; 2, full moon; 3, 3^rd^ quarter moon; 4, new moon; empty geometry shape, day-time; dark-filled geometry shape, night-time; square, flood tide; circle, ebb tide; DO, dissolved oxygen concentration; CY, *Cyanea* sp.; PP, *Phyllorhiza punctata*; LR, *Lobonemoides robustus*; RE, *Rhopilema esculentum*; RH, *Rhopilema hispidum*; CH, *Chrysaora chinensis*; AF, *Acromitus flagellatus*; LM, *Lychnorhiza malayensis*.

The relationship between the environmental parameters retained by DistLM and community structure is visualised in the constrained dbRDA ordination triplot (Fig. 5), with the first two axes explaining 94.7% of the fitted variance. Temporal differentiation in community structure was observed along the first dbRDA axis. The scyphozoan community could generally be discriminated into the wet (W) and dry (D) period community on the negative and positive side of the ordination plot, respectively. Among the species, *C. chinensis* (CH) was strongly associated with higher salinity and dissolved oxygen, and was more abundant during the dry period especially during the full and new moon (D2, D4) when the water temperature appeared warmer. On the other hand, *P. punctata* (PP), *R. esculentum* (RE), *L. malayensis* (LM), and *A. flagellatus* (AF) which were more abundant during the wet period, were apparently associated with lower salinity and DO. *Cyanea* sp. (CY) and *R. hispidum* (RH) were not associated with either the dry or wet periods, and their abundances were independent of the salinity and dissolved oxygen gradients. However, *Cyanea* sp. was associated with warmer temperatures and more turbid waters, while the opposite was observed for *R. hispidum*. Generally, the occurrence and abundance of the sampled scyphozoans in this study show no obvious pattern as a result of moon phase, diel and tidal cycle.

## Discussion

### Diversity and community structure of scyphozoan jellyfish

This study is among a few comprehensive studies conducted in tropical coastal waters to investigate the temporal community structure and abundance of scyphozoan jellyfish and how they relate to environmental conditions. The eight scyphozoan species collected from Klang Strait represents approximately 40% of the estimated Malaysian scyphozoan species richness (∼20 verified species; Syazwan et al., 2020). Scyphozoan diversity in the Klang Strait was comparably higher than those recorded in nearby tropical waters, for instance, the coastal waters of Penang (six species; Kwang, Yahya & Talib, 2009), Perak (three species; Chung, 2012), Surabaya, Indonesia (five genera; Saptarini, Aunurohim & Hayati, 2011) and the inner Gulf of Thailand (six species; Tandavanitj, 2001). This can be partly attributed to the difference in jellyfish sampling techniques. In particular, the other studies employed plankton net, scoop net, trawl net, or gill net, each covering a specific (limited) depth of the water column. In comparison, the present study used a bag net, a stationary gear that allows a greater collection of jellyfish across in the whole water column (2–10 m depth) at the same time. The higher number of species detected during the present study is also likely due to the longer extent of its temporal investigation (19 months). Previous studies in the Klang Strait had encountered six scyphozoan species in a 12 month-survey (Farrah-Azwa, 2016), and five scyphozoan species in a 2 months-survey (Azila & Chong, 2010).

### Effect of monsoon season and rainfall period

In the Klang Strait, scyphozoans were generally more abundant during the NEM (November–March) which coincided with the period of heavy rainfall (wetter period) but significantly decreased during the SWM (May–September) with less rainfall (drier period). High scyphozoan abundance during the wetter NEM is likely the consequence of increased food availability and primary production associated with the heavy rainfall. High precipitation and river inflows during this period are key factors for elevated primary production (*i.e.,* phytoplankton blooms) and increased heterotrophic bacterial productivity in the Klang Strait (Chong, King & Wolanski, 2005; Lee & Bong, 2008; Chew et al., 2015). Increased primary productivity could lead to high abundance of zooplankton especially copepods (Chew & Chong, 2011) that serve as the major food source for scyphozoans in the Klang Strait (Syazwan et al., 2021). Similar association between high jellyfish abundance and wet-rainy season has been observed in the Gulf of Mexico, where high precipitation and river inflows led to high nutrient loading and positively affected the productivity of jellyfish and their zooplankton preys (Robinson & Graham, 2013).

Temporal variations in scyphozoan occurrence in the Klang Strait are highly associated with the environmental dynamics regulated by the monsoon seasons and rainfall periods. The NEM especially during the wet period is characterized by significantly lower salinity, temperature, DO, and pH level. Among these parameters, salinity has been identified as the most important factor influencing the temporal pattern in scyphozoan community. It has been shown that changes in salinity could significantly affect population size, asexual reproduction and timing of appearance of many scyphozoans (Dawson, Martin & Penland, 2001; Purcell, 2005; Purcell, Uye & Lo, 2007; Holst & Jarms, 2010). An unprecedented increase in salinity (ca. 4.5) due to the El Niño phenomenon in Jellyfish Lake (Palau) had caused a decline in abundance of *Mastigias* sp., and detrimentally affected the body condition of *Aurelia* spp. (Dawson, Martin & Penland, 2001). In the present study, the mean salinity during SWM had risen by 2.3 relative to the NEM, while a larger increment of salinity by 5.8 was recorded at the Klang Strait’s main channel during SWM (Chong, 1993). Salinity increases during SWM, therefore, could be unfavourable to the local scyphozoans. Notably, *P. punctata* and *A. flagellatus*, which are known to have broad salinity tolerance, were more abundant during the wetter NEM when low salinities prevailed. Additionally, seasonal changes in salinity could be the major controlling factors of jellyfish prey (*e.g.*, copepod) abundance (Chew & Chong, 2011) in nearshore waters. Future study, therefore, is needed to clarify the relationship between seasonal scyphozoan abundance and salinity-induced-prey availability.

Temperature is another factor regulating the timing and duration of scyphozoan occurrences in the Klang Strait. Warm temperatures may enhance both asexual and sexual reproduction (Purcell, 2005; Kawahara et al., 2006; Liu et al., 2008), besides accelerating the growth of the pelagic stage of temperate scyphozoans (Møller & Riisgård, 2007; Rosa et al., 2013), although some species may respond differently to temperature changes (Fuchs et al., 2014). Thermal elevation may result in different consequences for tropical species that are already near their upper temperature limits (∼34–35 °C); hence, they may not be able to tolerate further warming (Dawson, Martin & Penland, 2001; Purcell, Uye & Lo, 2007; Brodeur et al., 2008). It has been shown that temperature at the study area can reach the maximum of 33.01 °C during the drier SWM (Chew et al., 2015). The warmer temperature during the SWM, to some extent, may exert adverse effects on scyphozoan population in the Klang Strait, although further studies are needed to investigate this aspect.

Increased scyphozoan abundance during the wetter NEM could be related to the general low concentration of DO (<4 mg/L) that is exacerbated by phytoplankton blooms and eutrophication in the Klang Strait (Lee & Bong, 2006; Lee & Bong, 2008; Lim, Lee & Kudo, 2015). Studies in other regions have shown that the pelagic and benthic stages of scyphozoans have a high tolerance to low-oxygen conditions (Purcell et al., 2001; Thuesen et al., 2005; Moriarty et al., 2012). Hypoxic water condition may benefit jellyfish feeding rates since prey mobility and escaping ability are hampered by low oxygen levels (Purcell et al., 2001; Decker, Breitburg & Purcell, 2004; Shoji et al., 2005). However, in areas experiencing eutrophication, such as in our study site, it is plausible that not all scyphozoan species can handle very low DO concentrations corresponding to hypoxic or anoxic conditions, at least in the polyp form (see Fernández-Alías et al., 2022; Enrique-Navarro et al., 2021). The complex interplay between nutrient inputs and DO concentration should be examined in future studies on characterizing scyphozoan abundance patterns.

The adaptive response by scyphozoans to the monsoonal driven environmental variability is species-specific. High abundance of *C. chinensis* during the drier SWM is consistent with findings from other studies. For instance, *C. chinensis* in Penang attained maximum abundance during the SWM months (Kwang, Yahya & Talib, 2009). *Chrysaora quinquecirrha* (Desor, 1848) in Chesapeake Bay (USA) usually increased in numbers when precipitation decreased, salinities were low, and temperatures were warmer (Purcell, 2005); these conditions bear close similarity to that of Klang Strait during the drier SWM months. Given that the abundance of *Cyanea* sp. is not apparently influenced by monsoon seasons, the species may have higher tolerance to the changing environmental conditions and thus able to dominate scyphozoan population. *Cyanea* species is known for its capability to withstand unfavourable physical conditions based on data from both laboratory experiments and field investigations from the Liaodong Bay, China (Dong et al., 2008).

Seasonal occurrence exhibited by individual scyphozoan species could be linked to the differences in timing of reproduction (Lucas, 2001; Lucas & Dawson, 2014). Analysis of the sexual reproductive status of two dominant species, *P. punctata* and *Cyanea* sp. (Table S2) indicates that their perennial occurrence could be the result of protracted spawning and juvenile recruitment throughout the year. Other species, *i.e., L. robustus*, *R. esculentum*, *L. malayensis*, and *A. flagellatus,* are only found during certain months in the Klang Strait. Seasonal abundance of these species is possibly related to the major period of mass strobilation, juvenile recruitment, sexual maturation and spawning in adults, that are likely regulated by the changing environmental conditions induced by the rainfall pattern (Lucas & Dawson, 2014).

### Effect of light periodicity and tidal cycle

Periodicity of light condition due to the diel cycle of solar radiation or lunar cycle of the waxing and waning moon (moon phases) has been reported to influence the composition and abundance of various marine invertebrates inhabiting shallow mudflats and mangroves estuaries in the Strait of Malacca (*e.g.*, Chew & Chong, 2011; Teoh, 2014; Ramarn, Chong & Hanamura, 2014). In the Klang Strait, the effect of diel cycle on scyphozoan abundance is evident during the wet period of the NEM, where scyphozoan abundances are generally higher during daytime rather than at night. *P. punctata*, which dominates the scyphozoan community during wet period (Table 2; Table S1), is known to be highly dependent on high light intensity since the species is nutritionally dependent on symbiotic zooxanthellae in their bodies (Bolton & Graham, 2004; Pitt, Koop & Rissik, 2005; Yap & Ong, 2012; Djeghri et al., 2019).

During the dry period of the SWM, the diel cycle has no effect on most scyphozoan species. One prominent exception is *Cyanea* sp. that increases in abundance during night-time especially during the spring tides of the dry period (Table S1). The *Cyanea* sp. population mainly consists of mature and spawning individuals during this period (Table S2). Spawning activities in many jellyfish species are inherently linked to diel periodicity of light (Purcell & Madin, 1991; Graham, Pagès & Hamner, 2001). Moon light intensity has been reported to have an impact on the spawning of fish (Ikegami et al., 2014; Krumme et al., 2015), invertebrates (Bentley, Olive & Last, 1999; Camargo, Van Vooren & Sorgeloos, 2002), and local shrimp species (Ramarn, Chong & Hanamura, 2014). Although it is unclear how the joint effect of night-time and moon light elicits spawning response in the scyphozoan, predation could be one reason. It has been reported that predatory fishes in the Klang Strait’s mudflat are mainly found during daylight (Lee, Chong & Yurimoto, 2016). Therefore, *Cyanea* sp. may aggregate in high abundance at night to spawn as a way to minimize predation on newly released eggs and larvae by visual predators (*e.g.*, larvae-eating fish).

Although our study did not examine the unique effect of tidal cycle, total scyphozoan abundance is comparatively higher during spring, flood tide than during neap, ebb tide events. Like juvenile fishes and other invertebrates in the area (Lee, Chong & Yurimoto, 2016), scyphozoans likely rely on the flood tide current as a passive transport mechanism (Gordon & Seymour, 2009; Albert, 2009; Albert, 2011; Fossette et al., 2015) to access their feeding and reproduction grounds in the inshore habitats, such as coastal mangrove habitats and coastal intertidal mudflats. Scyphozoan horizontal transport by swift flood current is particularly pronounced under spring tide condition due to the increased water level and stronger water current (Chong, King & Wolanski, 2005), thus explaining their higher catches during these tides.

One possible explanation for the lower catches during ebb tide is that scyphozoans could engage in vertical migration to the sea bottom to avoid offshore advection (by ebb tides; see Fig. 1) thus maintaining their dense aggregations near the coastline. Movement against prevailing currents and vertical migration in response to tidal exchanges have been reported for many jellyfish species (Magome et al., 2007; Albert, 2007; Albert, 2011; Fossette et al., 2015), especially by larger species or individuals due to their stronger swimming ability (Pitt & Kingsford, 2000; Fossette et al., 2015). *Cyanea* sp., *P. punctata*, and *L. robustus*, which dominated the scyphozoan catches in the Klang Strait are likely able to actively swim against the ebb current due to the larger body sizes (300–500 mm). In contrast, *A. flagellatus* and *L. malayensis* with the smaller body sizes (100–200 mm), could have been swept in the offshore direction by the strong spring-ebb current and thus sampled in relatively lower abundance.

### Limitations of study

The effects of biological factors such as prey availability, predators, competition and reproduction on scyphozoans abundance in Klang Strait are not within the scope of the present study. Various studies elsewhere, however, have shown that interactions between these biotic factors and abiotic factors (*i.e.,* environmental parameters and physical processes) are important in shaping temporal jellyfish community structure and abundance (Lucas & Dawson, 2014; Milisenda et al., 2018a; Milisenda et al., 2018b). Nevertheless, the plausible effects of some of these biological factors could be alluded albeit indirectly in the present study. Turbid water condition during spring tide has been associated with increased food availability (Chong, King & Wolanski, 2005; Nelson, 2005). The strong currents during spring-flood tide may enhance scyphozoan feeding success *via* increased encounter rates between scyphozoans and their zooplankton preys, while the highly turbid waters may reduce competition pressure from other visual zooplankton feeders (Arai, 1997). Higher scyphozoan abundance during the spring flood tide of dry period also could be linked to sexual reproduction and larvae dispersion strategy. The dominant species during the dry period, *Cyanea* sp., primarily comprised matured and spawning individuals. Formation of dense aggregations of these mature individuals, aided by the effect of strong spring-flood tide current, could facilitate spawning and fertilization success (Purcell et al., 2000; Graham, Pagès & Hamner, 2001; Lucas, 2001; Hamner & Dawson, 2009). Furthermore, the highly turbid waters during spring tide may be favourable for gamete fertilization and larval dispersion *via* reduced visibility of newly released eggs and larvae to potential predators. High tidal amplitudes and swift currents under spring-flood tide may also allow scyphozoans to disperse their larvae over a larger spatial scale, increasing accessibility to suitable habitats in the Klang Strait for the settling polyps.

The current study covered only the medusa stage of jellyfish, which is the dominant and familiar form. How or whether the benthic polyps and ephyrae in Klang Strait are similarly affected by the same set of physical factors is unknown. We had never been able to sample the polyps nor identify their natural settlement grounds on natural littoral vegetation and artificial structures including fishing stakes, fish cages and jetties. Future life-cycle studies will require more dedicated samplings, including the ephyrae using suitable plankton nets. Additionally, a minimum of 10 consecutive years of sampling would be ideal to characterize interannual variability, which can be very high (Condon et al., 2013). Such longer-term studies would allow in-depth examination of joint effects of periodicity, *e.g.*, diel and tidal cycles, on swarming behavior of species such as *P. punctata* and the commercial species including *L. robustus* and *R. esculentum,* which can in turn inform development of precautionary systems for users of beach areas or fishermen.

## Conclusions

This study shows that the temporal variation in the abundance and species composition of scyphozoan jellyfish in Klang Strait is driven by changes in salinity, DO, temperature, turbidity and pH in relation to the regional monsoon and local hydrographic regimes. Eight species found during the regular monthly sampling were also recorded during the intensive 24-hour sampling at both the dry and wet periods of the monsoon, with *P. punctata* and *Cyanea* sp. consistently being the dominant species. Generally, our findings corroborate with other studies that reported fluctuating scyphozoan population sizes over wide time scales, ranging from years to less than an hour (Condon et al., 2013). Our findings also challenge some beliefs that have been settled among the jellyfish scientist community for a couple of decades, that climate change might not benefit scyphozoan jellyfish species since there might be an upper temperature tolerance limit (Frolova, Retchless & Miglietta, 2024; Holst, 2012). While this could be particularly evident for winter species, this study is one of the first that finds a negative relationship between tropical jellyfish abundance and temperature. However, the asymmetrical response of the scyphozoan species demonstrated in this study suggest that future ecological models should consider them independently, rather that gathering them all into a single group.

The baseline information on seasonal or temporal abundance of jellyfish is useful for mitigating jellyfish impacts on human health and beach safety; for instance, an early warning system and emergency response to envenomation threats due to *C. chinensis*, *Cyanea* sp. and *R. hispidum* could be developed accordingly. Similarly, any serious harm to the operation of the Kapar power station could be avoided or managed using established mitigation methods (see Azila & Chong, 2010). Two commercially-valuable scyphozoan species in the Klang Strait, *L. robustus* and *R. esculentum* are revealed to be highly seasonal in their occurrence and abundance; fishery harvests and effective management of these species could be rationally implemented to take full advantage of their massive numbers, *e.g.*, based on an open- or closed season strategy that may be useful to avoid or reduce the damage of fishing gears and boat engines.

## Supplemental Information

10.7717/peerj.18483/supp-1 Supplemental Information 1 Percentage contributions of scyphozoan species to observed similarities (SIMPER) at all levels between monsoon season (SWM/NEM+IN), and within and across two nested factors (nested factors in brackets), namely period(moon) and diel(tide) Species significant contributions (% *δ* i > 3%, *δi*/*SD* > 1) to similarity are in bold. SWM, southwest monsoon; NEM+IN, northeast and inter-monsoon; Dry, dry period; Wet, wet period; Neap, neap tide (1st quarter and 3rd quarter moon); Spring, spring tide (full and new moon); D, day; N, night; F, flood tide; E, ebb tide; species: CY, *Cyanea* sp.; PP, *Phyllorhiza punctata*; LR, *Lobonemoides robustus*; RE, *Rhopilema esculentum*; RH, *Rhopilema hispidum*; CH, *Chrysaora chinensis*; AF, *Acromitus flagellatus*; LM, *Lychnorhiza malayensis*.

10.7717/peerj.18483/supp-2 Supplemental Information 2 Monthly mean CPUE (no. of individual/net) of two dominant scyphozoan species in the Klang Strait, *Phyllorhiza punctata* and *Cyanea* sp. based on life history stages (immature and mature medusa) Jellyfish was collected using the bag net from June 2010 to December 2011, covering different monsoon seasons: northeast monsoon (NEM), southwest monsoon (SWM), and inter-monsoon period (IN).

10.7717/peerj.18483/supp-3 Supplemental Information 3 Dataset of environmental parameters and scyphozoan abundance (CPUE) between monsoon seasons (monthly sampling routine) and rainfall periods (diel sampling series) in the Klang Strait CPUE, No. of individuals/net; Dry, dry period; Wet, wet period; 1Q, 1st quarter moon; 3Q, 3rd quarter moon; FM, full moon; NM, new moon; D, day; N, night; F, flood tide; E, ebb tide.

## Additional Information and Declarations

### Data Availability

The following information was supplied regarding data availability: The data is available at Zenodo: Syazwan, W. M. (2024). Temporal Drivers of Abundance and Community Structure of Scyphozoan Jellyfish [Data set]. Zenodo. https://doi.org/10.5281/zenodo.13173167.
