# Supplementary material for: Temporal drivers of abundance and community structure of scyphozoan jellyfish in tropical coastal waters
Source: PeerJ. 2025 Jan 16;13:e18483. doi: 10.7717/peerj.18483 (PMC11742249; doi:10.7717/peerj.18483)
Supplement: Supplemental Information 1 — Species significant contributions (% δ i > 3%, δi/SD > 1) to similarity are in bold. SWM, southwest monsoon; NEM+IN, northeast and inter-monsoon; Dry, dry period; Wet, wet period; Neap, neap tide (1st quarter and 3rd quarter moon); Spring, spring tide (full and new moon); D, day; N, night; F, flood tide; E, ebb tide; species: CY, Cyanea sp.; PP, Phyllorhiza punctata; LR, Lobonemoides robustus; RE, Rhopilema esculentum; RH, Rhopilema hispidum; CH, Chrysaora chinensis; AF, Acromitus flagellatus; LM, Lychnorhiza malayensis. [file peerj-13-18483-s001.pdf]

Table S1: Percentage contributions of scyphozoan species to observed similarities (SIMPER) at all levels between monsoon season (SWM/NEM+IN), and within and across two nested factors (nested factors in brackets), namely period(moon) and diel(tide).

Species significant contributions (%δi > 3%, δi/SD > 1) to similarity are in bold. SWM = southwest monsoon; NEM+IN = inter- and northeast monsoon; Dry = dry period; Wet = wet period; Neap = neap tide (1st quarter and 3rd quarter moon); Spring = spring tide (full and new moon); D = day; N = night; F = flood tide; E = ebb tide; species: CY = *Cyanea* sp.; PP = *Phyllorhiza punctata*; LR = *Lobonemoides robustus*; RE = *Rhopilema esculentum*; RH = *Rhopilema hispidum*; CH = *Chrysaora chinensis*; AF = *Acromitus flagellatus*; LM = *Lychnorhiza malayensis*.

Source of variation	Level of variation	Average similarity	% of species contribution to similarity							
			CY	PP	LR	RE	RH	CH	AF	LM
Monsoon	SWM	51.0	**57.3**	15.0	27.0	0.2	0.4	0.2	0.0	0.0
	NEM+IN	49.8	29.3	**59.9**	3.0	6.8	0.3	0.3	0.1	0.3
Period(Moon)	DryNeap	40.4	**74.2**	12.7	9.5	0.0	0.4	2.3	0.0	1.0
	DrySpring	48.0	**84.2**	2.4	9.7	0.4	0.0	2.1	0.2	1.0
	WetNeap	59.9	11.9	**61.0**	6.2	16.5	0.1	0.1	0.0	4.3
	WetSpring	61.4	**27.6**	**61.2**	1.6	4.6	0.1	0.3	0.7	3.9
Diel(Tide)	DF	44.8	**51.0**	32.1	12.6	2.8	0.0	0.8	0.1	0.6
	DE	36.5	**49.2**	22.2	7.4	7.4	0.3	1.9	1.8	9.8
	NF	45.5	**59.4**	25.1	11.5	2.7	0.1	0.3	0.0	1.0
	NE	44.7	**58.4**	33.8	1.0	1.8	0.1	1.8	0.2	3.0
Period(Moon)	**Dry(Neap)**									
x	DF	43.5	**61.2**	22.9	13.7	0.0	0.0	2.1	0.0	0.0
Diel(Tide)	DE	59.1	**87.5**	2.8	8.7	0.0	0.4	0.3	0.0	0.4
	NF	27.5	68.9	13.5	4.8	0.0	0.9	3.1	0.0	8.9
	NE	33.4	77.4	14.3	4.8	0.0	0.0	3.5	0.0	0.0
	**Dry(Spring)**									
	DF	61.4	**80.1**	2.6	16.4	0.0	0.0	0.9	0.0	0.0
	DE	27.1	59.6	0.0	10.2	4.5	0.0	8.8	6.0	11.0
	NF	68.9	**83.9**	2.4	13.5	0.1	0.2	0.0	0.0	0.0
	NE	58.6	**92.0**	3.1	0.8	0.0	0.0	2.6	0.0	1.5
	**Wet(Neap)**									
	DF	70.3	7.0	**55.3**	9.1	**27.6**	0.0	0.0	0.0	0.9
	DE	56.4	18.9	**34.4**	7.5	**25.3**	0.0	0.3	0.0	13.7
	NF	69.9	6.6	**71.5**	6.9	11.5	0.0	0.0	0.0	3.6
	NE	61.1	13.5	**80.3**	0.7	3.6	0.3	0.2	0.0	1.4
	**Wet(Spring)**									
	DF	60.6	**26.2**	**67.2**	2.6	1.5	0.0	0.4	0.3	1.8
	DE	66.1	**30.8**	**50.5**	2.0	5.6	0.2	0.4	1.7	8.8
	NF	70.6	**28.7**	**61.5**	3.1	6.7	0.0	0.0	0.0	0.0
	NE	62.5	**25.0**	**63.9**	0.1	5.2	0.0	0.4	0.5	5.0
