# Supplementary material for: Temporal drivers of abundance and community structure of scyphozoan jellyfish in tropical coastal waters
Source: PeerJ. 2025 Jan 16;13:e18483. doi: 10.7717/peerj.18483 (PMC11742249; doi:10.7717/peerj.18483)
Supplement: Supplemental Information 2 — Jellyfish was collected using the bag net from June 2010 to December 2011, covering different monsoon seasons: northeast monsoon (NEM), southwest monsoon (SWM), and inter-monsoon period (IN). [file peerj-13-18483-s002.pdf]

Table S2: Monthly mean CPUE (no. of individual/net) of two dominant scyphozoan species in the Klang Strait, *Phyllorhiza punctata* and *Cyanea* sp. based on life history stages (immature and mature medusa).

Jellyfish was collected using the bag net from June 2010 to December 2011, covering different monsoon seasons: northeast monsoon (NEM), southwest monsoon (SWM), and inter-monsoon period (IN).

Year	Monsoon	Month	*Phyllorhiza punctata*		*Cyanea* sp.	
			Immature	Mature	Immature	Mature
2010	SWM	June	0.00	1.00	0.46	1.62
	SWM	July	0.08	0.00	0.00	1.00
	SWM	August	0.00	0.29	0.64	1.57
	SWM	September	0.00	2.00	0.00	0.80
	IN	October	0.40	1.75	0.00	0.38
	NEM	November	0.88	1.80	0.40	0.20
	NEM	December	7.65	1.55	1.30	0.60
2011	NEM	January	3.80	10.80	0.00	0.30
	NEM	February	9.50	8.56	7.94	1.89
	NEM	March	1.22	0.50	2.33	4.83
	IN	April	0.00	0.82	3.47	0.53
	SWM	May	0.71	1.45	2.35	1.35
	SWM	June	0.25	4.21	0.86	1.29
	SWM	July	2.21	0.55	1.90	2.50
	SWM	August	0.00	0.00	2.13	3.63
	SWM	September	0.00	0.11	1.26	1.05
	IN	October	0.00	0.80	0.33	0.20
	NEM	November	0.40	6.07	2.71	0.93
	NEM	December	6.36	0.50	1.95	0.15
